# Coping strategies of neurosurgeons in response to major surgical complications: A nationwide questionnaire assessment in Germany

**DOI:** 10.1016/j.bas.2026.106258

**Published:** 2026-08-05

**Authors:** Lisa Schock, Matthias Mehdorn, Anne-Sophie Mehdorn, Mathias Wirth, Philipp Dammann, Ulrich Sure, Laurèl Rauschenbach

**Affiliations:** a Department of Neurosurgery and Spine Surgery, University Hospital Essen, University of Duisburg-Essen, Essen, Germany; b Center for Translational Neuro- and Behavioral Sciences (C-TNBS), University of Duisburg-Essen, Essen, Germany; c German Cancer Consortium (DKTK) Partner Site, University Hospital Essen, University of Duisburg-Essen, Essen, Germany; d Department of Visceral, Transplantation, Thoracic und Vascular Surgery, University Hospital Leipzig, Leipzig, Germany; e Department of General, Abdominal, Thoracic, Transplantation and Pediatric Surgery, University Hospital Schleswig-Holstein, Campus Kiel, Kiel, Germany; f Department of Systematic Theology/Ethics, University of Bern, Bern, Switzerland

**Keywords:** Neurosurgical complications, Second victim, Coping strategies, Error culture, Institutional support

## Abstract

**Introduction:**

This study aimed to explore the emotional and psychological impact of major surgical complications on neurosurgeons in Germany, focusing on identifying coping strategies, perceived barriers to support, and the influence of institutional culture – particularly in the context of the "second victim" phenomenon.

**Research question:**

How do neurosurgeons in Germany handle major complications, and which barriers limit access to psychological support?

**Material and methods:**

In this cross-sectional study, a nationwide, anonymous online survey was conducted among neurosurgeons working at university departments across Germany. The survey included 18 items assessing demographics, exposure to high-risk procedures, emotional responses to Clavien-Dindo Grade IV/V complications, coping mechanisms, and access to institutional support. Data from 110 completed questionnaires were analyzed using descriptive statistics.

**Results:**

Eighty percent of participants reported experiencing a major complication, with 60% still being emotionally affected. High levels of guilt and self-doubt were common. The most frequent coping strategies were proactive behavior (62.4%), rumination (57.8%) and seeking social support (60.6%). Only 26.4% reported to have had access to formal psychological support – although 82.7% of those without access considered it necessary. Maladaptive coping was more prevalent in low-tolerance error cultures, and early-career surgeons were more likely to externalize blame.

**Discussion and conclusion:**

Major complications considerably impact neurosurgeons’ mental health. Yet, institutional support remains insufficient. The findings call for mandatory, confidential psychological support, structured debriefings, and a cultural shift toward non-punitive, learning-oriented environments, supported by medical ethics, as a key instrument of cultural change to safeguard both surgeons' well-being and patient safety.

## Highlights

• Eighty percent of neurosurgeons experienced major complications.
• Proactive behavior, rumination and social support are dominant coping strategies.
• Only 26% had access to formal psychological support.
• Low error tolerance is linked to maladaptive coping behaviors.
• Mandatory debriefings and confidential support are urgently needed.

## 1 Introduction

Complications are an inherent and unavoidable aspect of neurosurgical practice, with significant implications for both patient outcomes and the well-being of surgical teams. Recent studies have highlighted the high incidence of postoperative complications in neurosurgery, with one prospective observational study reporting a complication rate of 21.6% among 963 patients undergoing cranial or spinal procedures – higher in cranial surgery (26.6%) than in spinal surgery (17.5%; Boissonneau et al., 2023). Infection emerged as the most common complication, and postoperative mortality reached 4.9% in the cranial group and 1.7% in the spinal group. These figures underscore the high-stakes nature of neurosurgical interventions, where even minor deviations can lead to severe patient harm or death.

The emotional and psychological toll of such adverse events extends beyond the patient and their family, profoundly affecting the surgeons involved. A nationwide survey of neurosurgeons in the US revealed that 58.4% had experienced a complication resulting in a patient's death, while 80.3% and 79.6% reported complications causing short-term and long-term harm, respectively (Piper et al., 2026). These events had a significant or profound impact on the emotional well-being of 55.6% and 38.0% of respondents, respectively. The term 'second victim' refers to healthcare professionals who experience psychological and emotional distress following an adverse event, whether or not the event was preventable. While the phenomenon is often associated with medical errors, it also applies to unexpected complications arising from complex surgical procedures, which are inherent risks of high-stakes interventions. This aligns with the broader definition of the second victim phenomenon as described in the literature (Seys et al., 2013; Scott et al., 2009; Infantes et al., 2025). Evidence from multiple surgical specialties confirms its prevalence and severity. In a study of plastic surgery residents, 88% reported being involved in a medical error, and 86.5% experienced emotional sequelae such as guilt, anxiety, and insomnia (Khansa et al., 2022). Similarly, in obstetrics and gynecology, 90% of surgeons reported that complications causing poor patient outcomes led to significant distress, with younger surgeons and women being more likely to report impacts on mental health, sleep, and physical well-being (Collings et al., 2025). The Boston Intraoperative Adverse Events Surgeons' Attitude (BISA) Study further confirmed this pattern: 84% of surgeons reported anxiety, guilt, sadness, or shame after an intraoperative adverse event (IAE), with emotional distress being particularly acute when the event was attributed to a surgeon's error (Han et al., 2017).

Coping mechanisms vary widely, with humor and intellectualization being the most commonly reported strategies among neurosurgeons (Piper et al., 2026). However, maladaptive coping mechanisms – such as avoidance or emotional suppression – are strongly associated with negative career outcomes: surgeons using such strategies are 5.84 times more likely to consider leaving the profession and 3.08 times more likely to limit their practice in the short term (Piper et al., 2026). A pattern of maladaptive coping may contribute to the elevated rates of alcohol abuse and dependence observed in the surgical workforce, with 15.4% of surveyed US surgeons meeting criteria for such disorders. These disorders are particularly observed among younger, female, and burned-out surgeons and those reporting a major medical error in the previous three months (Oreskovich et al., 2012).

Despite the widespread nature of this phenomenon, support remains inconsistent. In the BISA study, colleagues were the most helpful support system (42%) (Han et al., 2017). In the plastic surgery resident cohort, only 24.3% received emotional support after an adverse event, and while peers were the most valued source of support, institutional structures to facilitate this were lacking (Khansa et al., 2022). Similarly, in obstetrics and gynecology, female surgeons and those with less than 15 years of experience reported lower willingness to interact with colleagues after complications (Collings et al., 2025). A point prevalence study of visceral surgeons in German university hospitals found that 71.6% of surgeons without professional support expressed a desire for it (Mehdorn et al., 2024). While peer support and confiding in senior colleagues are frequently used, cultural barriers such as a "macho" attitude and poor team communication often prevent surgeons from seeking help (Infantes et al., 2025). The most desired form of support – discussing incidents with an attending physician – was endorsed by 97.4% of surgical trainees in one study, highlighting a clear unmet need for structured, accessible, and stigma-free support systems (Ginzberg et al., 2024). The analysis of factors influencing moral distress among healthcare professionals suggests the importance of various organizational culture aspects (Thomas et al., 2024).

In the present study, we aimed to shed light on feelings, concerns and coping strategies of German neurosurgeons facing major surgical complications through an anonymous nationwide online questionnaire assessment. Furthermore, structural components such as hierarchy and error tolerance policy – the extent to which institutions foster a non-punitive, learning-oriented environment for discussing errors and complications – as well as professional management and support related to surgical complications were considered.

## 2 Methods

### 2.1 Sample

We administered a web-based questionnaire using the online survey platform Lime Survey (https://www.uni-due.de/zim/services/limesurvey.php) to assess neurosurgeons' perspectives and practices regarding experiences with surgical complications and coping strategies. Recruitment was conducted in two phases. First, an initial call for participation was sent via the email distribution list of the German Society of Neurosurgery (Deutsche Gesellschaft für Neurochirurgie, DGNC, 2008 members). Second, a targeted follow-up invitation was sent to the email addresses of male and female neurosurgeons affiliated with university hospitals across Germany. The survey was open for ten weeks starting in January 2026, allowing participants to complete the questionnaire at their convenience. Participation was voluntary and anonymous, and informed consent was obtained prior to initiation of the survey. Data were collected and stored securely in accordance with data protection regulations.

Ethical approval was obtained from the medical faculty of the Christian-Albrechts-Universität zu Kiel (D 462/25).

### 2.2 Questionnaire

The questionnaire was adapted from the questionnaire of Mehdorn et al. (Mehdorn et al., 2024) about surgical complications in visceral surgery and comprised 18 items across four thematic areas: (1) demographic and professional background; (2) exposure to high-risk procedures; (3) emotional and psychological responses to severe complications (Clavien-Dindo Grade IV/V; Dindo et al., 2004); and (4) organizational support and coping strategies. Questions covered gender, age group, years of experience, professional position, subspecialty focus, and frequency of performing high-risk elective surgeries (e.g., vascular malformations, eloquent tumor resections), excluding emergency interventions. Participants were asked to report any prior experience with life-threatening complications or patient death, as well as whether they currently feel emotionally affected.

Emotional reactions, such as guilt, self-doubt, fear, anger, helplessness, and depersonalization, were assessed using a 10-point Likert scale (1 = never; 10 = always). Participants also indicated their perceived personal responsibility for adverse outcomes and reported their use of coping mechanisms, including seeking social support, avoidance, substance use, and proactive learning.

Organizational aspects included departmental hierarchy, openness to discussing errors, availability of formal support, and participation in structured debriefings (e.g., morbidity and mortality conferences). Questions about psychological support were conditional (i.e., participants were only shown these questions if they reported that support was unavailable or available), ensuring relevance.

All questions were designed to elicit a single response, except for the item assessing coping strategies, for which multiple selections were allowed. Cookie-based prevention of duplicate responses was implemented. To ensure comprehensive coverage of the multifaceted nature of the topic, the instrument was developed through a multidisciplinary collaboration between visceral surgeons, neurosurgeons, a neuropsychologist, and a medical ethicist.

The complete questionnaire can be found in Supplementary Material.

### 2.3 Data analysis

The survey data were exported from the online platform (Lime Survey) in DAT format and imported into IBM SPSS Statistics (version 31.0.2.0) for analysis. Descriptive statistics were computed to assess the distribution of demographic and professional characteristics, as well as responses to Likert-scale and categorical items. Frequencies and percentages were used to summarize categorical variables, and measures of central tendency and dispersion were reported for continuous or ordinal data, as appropriate. The dichotomization of variables was conducted by means of the division of the 10-point Likert scale into two distinct halves. Missing data were handled using casewise deletion. Figures were generated using PRISM 10 software (version 10.4.1; GraphPad).

### 2.4 Artificial intelligence

Language editing of the manuscript was conducted with the assistance of the Chat AI tool provided by the University of Duisburg-Essen (https://www.uni-due.de/de/digitalisierung/ki-portal/chat-ai-login.php). All content was thoroughly reviewed, critically evaluated, and substantively verified by the authors.

## 3 Results

### 3.1 Sample characteristics and organizational structure

A total of 142 participants answered the questionnaire, 32 of which were incomplete. This resulted in 110 completed data sets. One item was missing for five participants, and five items were missing for two participants. The exact response rate cannot be calculated because of the call for participation via newsletter and email, as described in the Methods section.

Demographic and professional characteristics are described in Table 1.

**Table 1:** Demographic and professional characteristics of neurosurgery participants.

Category	Subcategory	Percentage
Sex	Male	74.5
	Female	25.5
Age group (years)	25-30	10.9
	31-40	33.6
	41-50	26.4
	51-67	29.1
Years of experience in neurosurgery	1-5	17.3
	6-10	20.0
	11-20	27.3
	More than 20	35.5
Professional position	Resident	24.5
	Specialist	9.1
	Senior Physician	38.2
	Section head	7.3
	Department chair	20.9
Main area of focus in neurosurgery	No specific focus	29.1
	Neurooncological surgery	28.2
	Neurovascular surgery	7.3
	Spinal surgery	15.5
	Neurotraumatology/neurointensive care	5.5
	Pediatric neurosurgery	5.5
	Functional neurosurgery, epilepsy surgery, or radiosurgery	7.3
	Peripheral nerve surgery	0.9
	Other	0.9

*Note.* N = 110.

A total of 17.3% of respondents strongly agreed with the statement *"The organization is strongly hierarchical"*, and 50.9% rated the statement 8 to 10 on a 10-point scale with 10 being strongest agreement; 65.4% of respondents indicated a higher level of agreement with the statement "*Errors are openly discussed and constructively addressed",* 34.6% reported a lower level of agreement.

### 3.2 Complications

The frequency with which participants performed elective procedures with a relevant risk of life-threatening complications or severe neurological deficits was as follows: 12.7% performed such procedures daily, 20.9% three times per week, 28.2% once per week, 13.6% twice per month, and 24.5% less than twice per month.

Of the participants, 80.9% reported having experienced complications of Clavien-Dindo grade IV or V – defined as life-threatening complications requiring intensive care or patient death – following a surgery for which they were the primary surgeon.

Experiencing complications in their role as primary surgeon emotionally affects 60.0% of the participants in their current position; 31.8% stated it does not, and 8.2% did not provide a response. A total of 55.5% reported feeling personally responsible for a complication or a patient's death, while 20.9% denied personal responsibility, and 23.6% were uncertain.

In Table 2a, Table 2b, worries and feelings associated to complications are depicted. Neurosurgeons reported high empathy for patients and relatives (median 8.00), but also strong feelings of guilt (7.00) and self-doubt (8.00). Concerns about legal consequences and being blamed by colleagues were moderate (median 5.00 and 4.00, respectively), while worries about reputation and quality criteria were higher (median 6.00). Anger and helplessness were reported at moderate levels (median 4.00), while depersonalization was rare (median 1.00).

**Table 2a:** Worries associated to major surgical complications.

Worries	Median	IQR
Empathy for the patient	8.00	3
Empathy for the relatives	8.00	3
Quality criteria	6.00	4
Reputation	6.00	5
Legal consequences	5.00	5
Being blamed by colleagues	4.00	5

*Note.* Answers were collected on a 10-point Likert scale from 1 to 10; IQR: interquartile range.

**Table 2b:** Feelings associated to major surgical complications.

Feelings	Median	IQR
Self-doubt	8.00	4
Guilt	7.00	4
Anxiety	4.00	4
Anger	4.00	5
Helplessness	4.00	4
Depersonalization	1.00	2

*Note.* Answers were collected on a 10-point Likert scale from 1 to 10; IQR: interquartile range.

### 3.3 Coping mechanisms

**Fig. 1:**
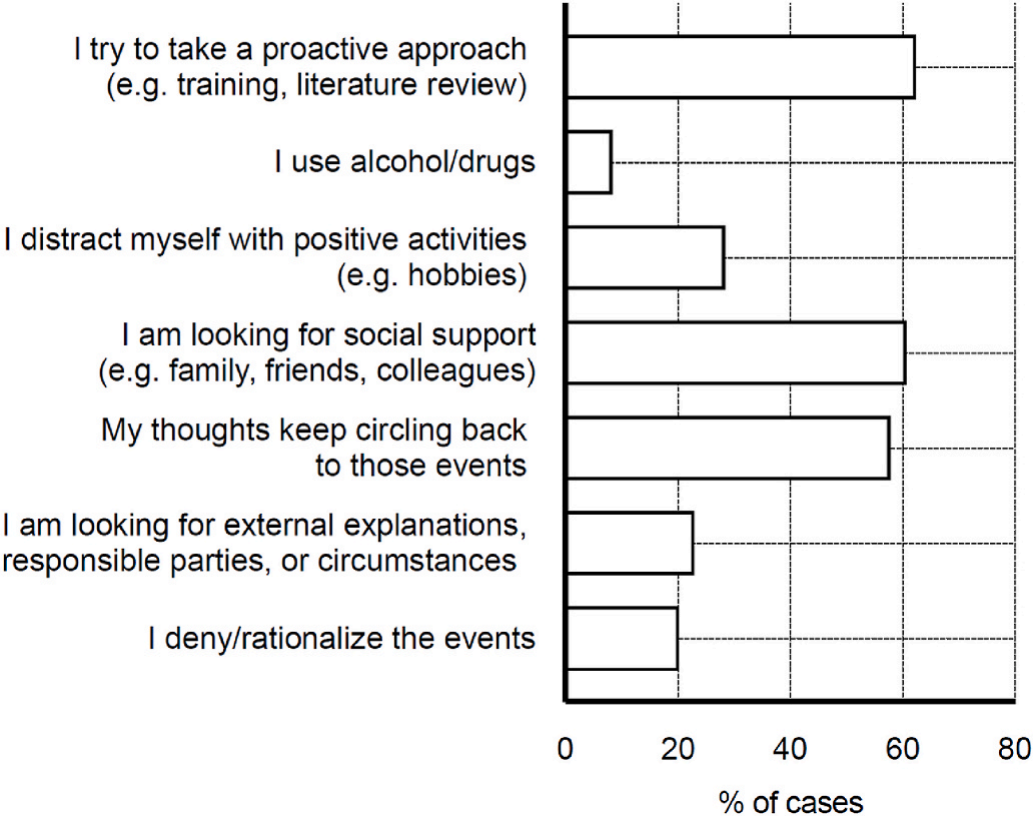
Coping strategies of neurosurgeons related to major complications *Note.* Answers are shown in percent of cases.

The most commonly reported coping strategies among neurosurgeons following a serious complication were rumination (57.8%) and seeking social support (60.6%), highlighting the central role of emotional processing and peer interaction in post-event recovery. Proactive coping, such as engaging in additional training or literature review, was also highly prevalent (62.4%). In contrast, maladaptive strategies, such as denial, rationalization, or substance (ab)use, were reported by fewer respondents (22.9%, 20.2%, and 8.3%, respectively), suggesting that these strategies are not dominant, yet still prevalent. Refer to Fig. 1.

**Fig. 2:**
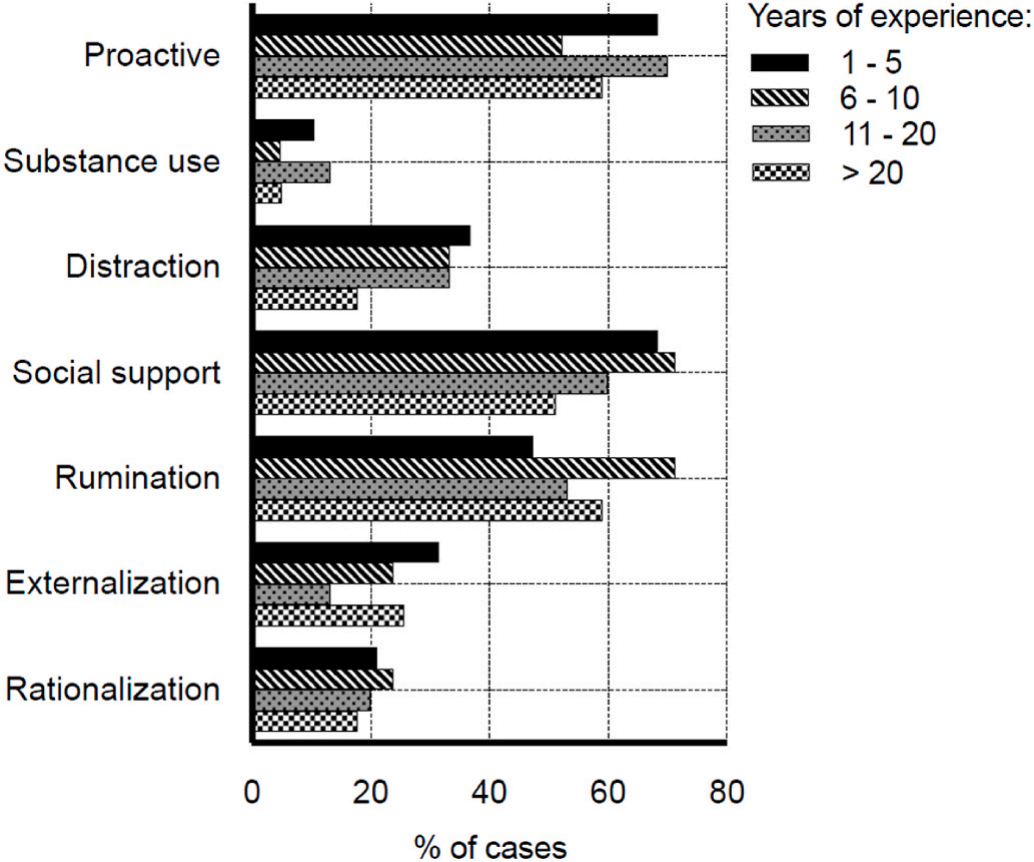
Coping strategies divided by amount of professional experience *Note.* Answers are shown in percent of cases.

As shown in Fig. 2, rumination (47.4%-71.4%) and seeking social support (51.3%-71.4%) were the most consistently reported coping strategies across all experience groups, with the highest rates among those with 6-10 years of practice. Proactive coping (e.g., training, literature review) was also prevalent, particularly among surgeons with 11-20 years of experience (70.0%), though slightly lower in early-career (68.4%) groups. In contrast, maladaptive strategies such as denial (17.9%-23.8%) and substance use (4.8%-13.3%) were relatively low, with substance use being more prominent in early career and 11-20 years experienced neurosurgeons. Notably, externalizing blame was most common among early-career surgeons (31.6%). Seeking positive activities was least pronounced in the late-career group (17.9%, more than 20 years).

**Fig. 3:**
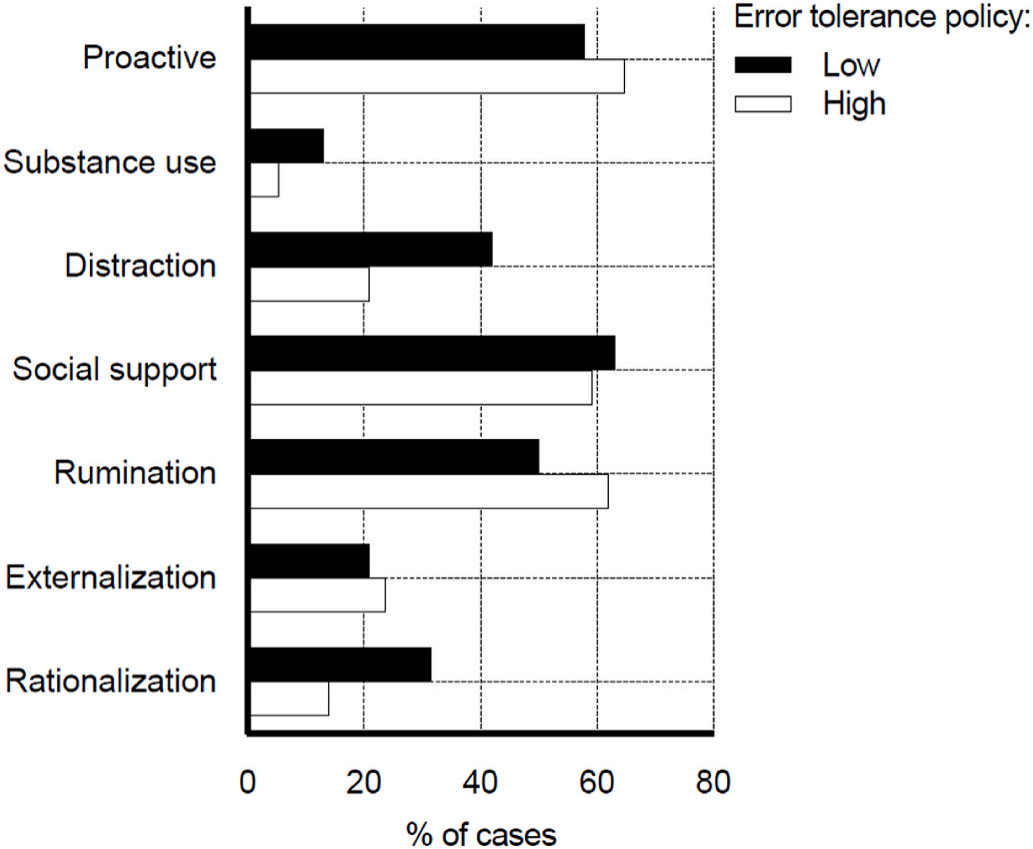
Coping strategies divided by ratings of high and low error tolerance policy *Note.* Answers are shown in percent of cases.

Surgeons in high-tolerance policy environments reported higher rates of rumination (62.0% vs. 50.0%) and proactive coping (64.8% vs. 57.9%) compared to those in low-tolerance settings. In contrast, denial and rationalization were more common in low-tolerance environments (31.6% vs. 14.1%), suggesting a potential shift toward avoidance under stricter accountability. Externalizing blame and seeking social support was similar across groups (21.1% vs. 23.9%; 63.2% vs. 59.2%). Notably, distraction through hobbies was more frequent in low-tolerance environments (42.1% vs. 21.1%), and substance use was higher in low-tolerance settings (13.2% vs. 5.6%), indicating possible differences in coping intensity or access to support. Compare Fig. 3.

### 3.4 Professional coping

In response to the question about structured forums for reflecting on (fatal) complications, 80.0% of participants reported that such conferences exist either within their department or in an interdisciplinary setting. Regarding professional support for managing psychological stress following adverse events, 26.4% of respondents stated that their employer offers such support.

When asked whether professional support should be offered in their institution, 82.7% (n = 67) answered affirmatively, 17.3% (n = 14) responded negatively. Of those who reported that professional support was available (n = 29), only 13.8% (n = 4) had ever utilized it, while 86.2% (n = 25) had not used it. Among those who had used professional support, 100.0% (n = 4) reported that it had helped them cope with the psychological burden following (fatal) complications.

## 4 Discussion

The present study provides critical insights into the emotional, psychological, and organizational landscape surrounding major surgical complications among German Academic Departments of Neurosurgery and their respective neurosurgeons. With a high prevalence of life-threatening complications (Clavien-Dindo Grade IV/V) reported by 80.9% of participants our findings confirm that adverse events are not rare exceptions but a routine, albeit distressing, component of the specialty. Our data demonstrate that 60.0% of neurosurgeons report ongoing emotional impact from past complications. Moreover, a substantial majority (55.5%) experience a sense of personal accountability following serious adverse events. In the context of the "second victim" syndrome (Seys et al., 2013; Scott et al., 2009; Infantes et al., 2025; Khansa et al., 2022; Collings et al., 2025; Han et al., 2017; Vernon et al., 2025), the profound emotional impact of such outcomes is highlighted, as is the need to treat the psychological well-being of surgeons as a legitimate and urgent concern within patient safety frameworks.

### 4.1 Emotional and psychological impact of complications

The emotional burden reported by neurosurgeons is multifaceted. High median scores for empathy toward patients and their families (8.00/10) reflect a deep professional commitment and emotional investment, which, while clinically valuable, also heightens vulnerability to psychological distress after adverse outcomes. This emotional resonance is mirrored in the elevated levels of guilt (7.00) and self-doubt (8.00), which are consistent with findings from other surgical disciplines. A multinational study of neurointerventionalists revealed that 64% exhibited symptoms consistent with possible post-traumatic stress disorder (PTSD) following major complications, with intrusion – such as persistent distressing thoughts about the event – being the dominant symptom domain. Notably, lack of institutional support was significantly associated with higher PTSD risk (Rai et al., 2025). Similarly, a phenomenological study in Nigeria found that 90.3% of surgical residents had personally experienced a medical error, leading to intense emotional distress, sleep disruption, and a pervasive sense of guilt, despite relying primarily on peer support from colleagues, highlighting systemic gaps in formal psychological safety structures (Balogun et al., 2023). In a broader perspective, a recent review emphasized that the consequences of adverse events extend far beyond the operating room, affecting surgeons’ mental and physical health, career satisfaction, and even professional behavior, with burnout and defensive medicine becoming common outcomes (Awuah et al., 2024).

Notably, concerns about legal consequences (median 5.00), reputation (6.00), and being blamed by colleagues (4.00) suggest that fear of professional repercussions plays a significant role in shaping post-event emotional processing. These findings resonate with the BISA study, where 50% of surgeons cited fear of litigation as a barrier to reporting adverse events (Han et al., 2017).

### 4.2 Coping strategies and their variation by experience and culture

The coping strategies employed by neurosurgeons reveal both resilience and vulnerability. Rumination (57.8%) and seeking social support (60.6%) were the most prevalent strategies. This is encouraging, as peer support might mitigate the long-term psychological impact of adverse events (Khansa et al., 2022). However, the high rate of rumination could be interpreted as a maladaptive coping strategy (Mehdorn et al., 2024). A total of 62.4% of neurosurgeons reported engaging in proactive learning after complications, suggesting a substantial, though not universal, interest in reflective practice. Yet, the relatively low use of formal psychological support despite strong demand points to a systemic failure in institutional response. A systematic review identified that surgeons commonly resort to limited peer discussion, physical activity, and creative outlets as coping mechanisms, while some turn to alcohol or substance use (Srinivasa et al., 2019). Similarly, a recent Irish cross-sectional study found that while 67% of surgeons reported their training did not prepare them for the personal impact of adverse events, they still found informal peer and family support beneficial (O'Meara et al., 2025).

Interestingly, coping patterns varied by professional experience. Early-career surgeons (1-5 years) were more likely to externalize blame (31.6%), a finding that may reflect immaturity in emotional regulation or a lack of psychological resilience. In contrast, surgeons with 11-20 years of experience showed the highest rates of proactive coping (70.0%), suggesting that experience may foster adaptive strategies over time. However, late-career surgeons (>20 years) reported the lowest engagement in positive activities (e.g. hobbies), which may signal emotional fatigue or disengagement – a red flag for long-term workforce sustainability. These age-related differences underscore the importance of developmentally appropriate support systems, tailored to the needs of trainees, mid-career surgeons, and senior clinicians alike (Fall et al., 2024; El Hechi et al., 2020). This is especially relevant in light of the finding that psychological instability seems to decrease and then increase over the course of a neurosurgeon's career (Kalywis et al., 2023).

One of most striking results of our study is the influence of organizational culture, particularly error tolerance policies. The organizational structure of the surveyed clinics and departments is perceived as highly hierarchical, and a constructive error culture is not fully established. Surgeons in high-tolerance environments tended to report higher rates of rumination and proactive coping, suggesting that a non-punitive, learning-oriented culture might partly foster psychological safety. Conversely, in low-tolerance settings, maladaptive strategies such as denial/rationalization (31.6% vs. 14.1%) and substance use (13.2% vs. 5.6%) were proportionally higher, possibly indicating that fear of blame drives avoidance and unhealthy coping. This finding is consistent with the notion that institutions prioritizing responsibility over learning contribute to the development of the second victim syndrome (Hsiao et al., 2025). The fact that distraction through hobbies was more common in low-tolerance environments (42.1%) further suggests that surgeons may resort to avoidance as a defense mechanism when they feel unsupported.

### 4.3 Institutional support and the gap between need and use

Despite the existence of morbidity and mortality (M&M) conferences in 80.0% of departments, the lack of formal psychological support – especially in the absence of structured debriefings or mental health resources – limits their effectiveness. M&M conferences, while valuable for quality improvement and often held because of certification regulations, are not designed to address the emotional needs of the surgeon. The integration of structured psychological debriefings could bridge this gap, providing a safe space for emotional processing, peer support, and professional reflection without fear of judgment (Eidt and Mannoia, 2024; Le et al., 2023).

Our findings also highlight a critical gap in institutional support systems in Germany. In negative accordance with German Academic Departments of Visceral Surgery where only 17.2% offered professional support, despite high demand (Mehdorn et al., 2024), the situation in neurosurgery appears similarly devastating with psychological support only being offered to 26.4%. This is particularly concerning given that younger and female surgeons seem to be more vulnerable concerning mental well-being (Collings et al., 2025; Mehdorn et al., 2024). Without targeted interventions, these disparities may contribute to gender imbalances in leadership and higher attrition rates among early-career neurosurgeons.

### 4.4 Implications for training, medical ethics, and future research

Furthermore, psychological safety training (Po, 2026) should be integrated into surgical education from early stages of training on. Incorporating simulation-based scenarios that address both technical challenges and emotional responses to complications can equip trainees with the tools to manage stress, reflect on their actions, and maintain professional well-being throughout their careers. Medical ethics, too, can be engaged as a resource in this context, as it encompasses dimensions relevant not only to patient ethics but also to professional ethics (Westarp et al., 2026). Enhanced ethical reflection within neurosurgery can help accompany the complex decision-making processes involved in high-risk interventions, strengthen their respective justifications, and thereby mitigate “moral distress” in the event of adverse outcomes.

While our cross-sectional data do not establish causality, they highlight a clear need for further research into the impact of institutional culture on surgeon well-being. Future longitudinal and interventional studies are warranted to evaluate whether structured psychological support and error-tolerant environments improve both mental health outcomes and patient safety. Targeted, evidence-based interventions, such as psychological debriefings and culture change initiatives, should be piloted and evaluated in academic neurosurgical centers.

Our study has several limitations. First, the response rate cannot be precisely calculated due to overlapping recruitment channels (newsletter and direct emails), which may introduce selection bias. Second, the sample was predominantly male (74.5%), which limits generalizability to female neurosurgeons and may reflect broader gender disparities in the field. Third, the cross-sectional design – as mentioned above –prevents causal inference. Fourth, self-reported data may be subject to recall or social desirability bias, particularly regarding the perceived need for psychological support. Respondents may overstate their willingness to use formal support due to perceived social expectations, even if actual utilization remains low. Moreover, participants could have contributed more than once when using different devices. Due to strict data protection regulations, it was not possible to link institutional affiliations to responses, which prevented the identification of potential clustering effects, possibly undermining the representativeness of the findings. Given the study design, inferential statistical testing is not appropriate. Therefore, only descriptive analyses can be conducted, and claims about group differences should be made cautiously. Finally, our findings are specific to German academic neurosurgery and may not be directly transferable to other healthcare systems, non-academic settings, or surgical specialties with different cultural or legal environments.

## 5 Conclusion

The findings of this study underscore the urgent need for systemic changes in how neurosurgical institutions respond to adverse events. Given that a majority of neurosurgeons report significant emotional distress following major complications, and that only a small fraction has access to formal psychological support, it is imperative that hospitals and academic centers institutionalize mandatory, confidential, and stigma-free psychological support programs. These programs should provide immediate access to trained mental health professionals, peer support networks, and structured post-event debriefings to help surgeons process their experiences in a safe and supportive environment. However, our findings reveal a critical paradox: support is often not utilized, even when it is available. This underscores the fact that access alone is insufficient. Awareness, trust, and cultural acceptance of psychological care remain key barriers. Therefore, targeted awareness campaigns within neurosurgical societies and systematic education on emotional distress, coping strategies, and available resources are urgently needed.

## Consent to participate

All participants gave electronic informed consent before participation in the study.

## Author contribution information

Conceptualization: LS; LR; Methodology: LS, MM, ASM, LR; Formal analysis and investigation: LS, LR; Data curation: LS, LR; Writing – original draft preparation: LS, MW, MM, ASM, LR; Writing – review and editing: all authors; Supervision: PD, US, LR; Resources: US.

## Data availability

Any data not published within the article will be shared in an anonymized manner at request from any qualified investigator.

## Ethics approval

The study was approved by the local ethics committee of the Christian-Albrechts-Universität zu Kiel (D 462/25) and was conducted according to the tenets of the Declaration of Helsinki.

## Consent to publish

The authors declare that they agree with the content of the manuscript and consent to publish.

## Funding

The authors received no specific funding for this work.

## Declaration of competing interest

The authors declare that they have no known competing financial interests or personal relationships that could have appeared to influence the work reported in this paper.
