# Supplementary material for: Coping strategies of neurosurgeons in response to major surgical complications: A nationwide questionnaire assessment in Germany
Source: Brain Spine. 2026 Aug 5;6:106258. doi: 10.1016/j.bas.2026.106258 (PMC13485496; doi:10.1016/j.bas.2026.106258)
Supplement: Supplementary file 1 [file mmc1.docx]

The psychological impact of serious surgical complications in neurosurgery – Questionnaire

Dear Colleagues,

Neurosurgery is a surgical discipline that demands extensive experience, knowledge of anatomy, and specialized surgical techniques from surgeons. Despite all modern methods, life-threatening complications – including the death of the patient – can still occur. The challenge lies not only in mastering surgical techniques and technical capabilities, but also in coping with complications that arise from one’s own actions. In this regard, everyone will have their own wealth of experience. Since the topic of coping with fatal patient outcomes and its impact on surgeons has not yet been sufficiently studied, we aim to investigate, as part of this exploratory survey, the subjective stress experienced by surgeons due to fatal patient outcomes and their coping strategies.

The survey takes only a few minutes. Thank you very much.

Demographic Information:

1. What is your gender? (Select one answer)

a)	Male

b)	Female

c)	Non-binary

2. How old are you? (Select one answer)

a)	25–30 years old

b)	31–40 years old

c)	41–50 years

d)	51–67 years

Qualifications and Work Environment:

3. How long have you been working in neurosurgery? (1 answer allowed)

a)	1–5 years

b)	6–10 years

c)	11–20 years

d)	More than 20 years

4. What is your position in your department? (1 answer allowed)

a)	Resident

b)	Board-certified specialist

c)	Senior physician

d)	Section head

e)	Department chair

5. What is your area of specialization within neurosurgery? (1 answer allowed)

a)	I do not (yet) have a specific area of specialization

b)	Neurovascular surgery

c)	Neuro-oncological surgery

d)	Neurotraumatology / Neurocritical care

e)	Spinal surgery

f)	Neuropediatric surgery

g)	Functional neurosurgery / Epilepsy surgery / Radiosurgery

h)	Peripheral nerve surgery

i)	Other

6. Which of the following applies to the organizational structure of your clinic / department? (Rate each on a Likert scale of 1–10)

a)	The organization is highly hierarchical

b)	Mistakes are openly addressed and dealt with constructively

7. How often do you perform elective procedures with a significant risk of life-threatening complications or severe neurological deficits, e.g., treatment of vascular malformations, resection of tumors in eloquent locations (emergency procedures are excluded)? (1 answer allowed)

a)	Daily

b)	3 times per week

c)	1 time per week

d)	2 times per month

e)	Less than 2 times per month

Dealing with Complications:

8. Have you ever experienced Clavie-Dindo Grade IV or V complications (Grade IV: life-threatening complications requiring intensive care; Grade V: death of the patient) in a patient following a surgical procedure for which you were the primary surgeon? (1 answer allowed)

a)	Yes

b)	No

9. Does this place an emotional burden on you in your current position? (1 answer allowed)

a)	Yes

b)	No

c)	No answer

10. If a life-threatening complication occurs in one of your patients, what are your greatest concerns? (on a Likert scale of 1–10 for each)

a)	I feel compassion for the patient

b)	I feel compassion for the family

c)	I am concerned about legal consequences

d)	I am concerned about quality standards

e)	I am afraid of being blamed by colleagues

f)	I am concerned about my reputation

11. Do you feel personally responsible for a patient’s complication or death? (1 answer allowed)

a)	Yes

b)	No

c)	Unsure

12. Have you experienced the following feelings following a patient’s complication or death? (on a Likert scale of 1–10 for each)

a)	Guilt

b)	Self-doubt

c)	Fear

d)	Anger

e)	Helplessness

f)	Depersonalization/derealization

13. How do you deal with such an event? Please select the answers that best apply. (Multiple answers allowed)

a)	I deny or rationalize the events

b)	I look for external explanations, people to blame, or circumstances

c)	My thoughts keep circling back to the events

d)	I seek social support (family/friends/colleagues)

e)	I distract myself with positive activities (hobbies, etc.)

f)	I consume alcohol/drugs

g)	I try to act proactively (training, literature review, etc.)

Support options:

14. Are there conferences in your department or in an interdisciplinary setting to address (fatal) complications? (1 answer allowed)

a)	Yes

b)	No

15. Does your employer offer professional support to help deal with such psychologically stressful situations? (1 answer allowed)

a)	Yes

b)	No

16. Should professional support services be available at your institution? (Appears only if question 15 was answered “No”; 1 answer allowed)

a)	Yes

b)	No

17. Have you ever utilized this professional support? (Appears only if question 15 was answered “Yes”; 1 answer allowed)

a)	Yes

b)	No

18. Did this professional help assist you in coping with the psychological stress following (fatal) complications? (Appears only if question 17 was answered with “Yes”; 1 answer allowed)

a)	Yes

b)	No
